# Longitudinal Evaluation of the QuantiFERON-TB Gold Plus Assay in Hospitalized COVID-19 Patients with a First Indeterminate Result: Resolution of Inflammation and Restoration of T-Lymphocyte Counts and Interferon-Gamma Production

**DOI:** 10.1128/spectrum.01858-22

**Published:** 2022-09-13

**Authors:** Grazia Alessio, Alessandra Imeneo, Andrea Di Lorenzo, Benedetta Rossi, Chiara Sorace, Mirko Compagno, Luigi Coppola, Laura Campogiani, Angela Maria Antonia Crea, Vincenzo Malagnino, Francesco Buccisano, Massimo Andreoni, Loredana Sarmati, Marco Iannetta

**Affiliations:** a Department of Systems Medicine, Tor Vergata University, Rome, Italy; b Department of Biomedicine and Prevention, Tor Vergata University, Rome, Italy; Quest Diagnostics Nichols Institute

**Keywords:** coronavirus, QuantiFERON, IGRA, tuberculosis, T-lymphocyte, SARS-CoV-2, interferons

## LETTER

Several studies report an increased rate of indeterminate QuantiFERON-TB Gold Plus (QFT-P) results in patients hospitalized because of severe Coronavirus Disease (COVID)-19, and this is due to peripheral blood T-lymphocyte depletion and dysfunction (1–6), which are known to be associated with COVID-19 disease severity and mortality (7–9). So far, there are no reports concerning QFT-P responses and relations with lymphocyte counts or inflammation markers in subjects who survived COVID-19 and received a previous indeterminate result after the acute phase of the disease.

For this reason, patients hospitalized because of COVID-19 from March 2020 to August 2021 in the Infectious Disease Unit of Policlinico Tor Vergata University Hospital in Rome who received an indeterminate QFT-P test during hospitalization were recalled for reassessment after recovery in the post-COVID outpatient clinic. Those who gave written informed consent were enrolled in the study. Collection tubes for the QFT-P test were purchased from Qiagen, and the interferon gamma (IFN-γ) levels were assessed via chemiluminescence immunoassay using the Liaison XL Analyzer. In a subgroup of patients, peripheral blood lymphocyte subsets were also reassessed. Demographics, clinical data, and laboratory data were collected. The study was approved by the local Ethics Committee (protocol number 125.21). All statistical analyses were performed using GraphPadPrism. Comparisons between groups were performed via a nonparametric test for paired data (Wilcoxon), and a *P* value of <0.05 was regarded as indicative of a statistically significant result.

36 patients were enrolled, and the sample presented with a median age of 58 (interquartile range [IQR]: 51 to 63) years and a prevalence of males (M/F: 24/12). Considering disease severity, 30 patients were classified as severe (needing high flux oxygen or noninvasive or invasive ventilation), and 6 were classified as nonsevere. 1 patient needed admission to the intensive care unit (ICU). The median Charlson comorbidity index was 2 (IQR:1 to 3) (Table 1).

**TABLE 1:** Demographic and clinical characteristics of the subjects included in the study^*a*^

Parameter	Total N = 36	QFT-P negative^*b*^ N = 34	QFT-P positive^*b*^ N = 2
Demographic			
Age	58 (51 to 63)	57 (50 to 63)	81 and 60
Sex (M/F)	23/13	22/12	1/1
Ethnicity	32 Caucasian 1 African 2 Asian 1 Hispanic	31 Caucasian 0 African 2 Asian 1 Hispanic	1 Caucasian 1 African 0 Asian 0 Hispanic
Charlson comorbidity index	2 (1 to 3)	2 (1 to 3)	6 and 1
COVID-19 hospitalization			
Length of hospital stay	15 (10 to 18)	15 (11 to 19)	10 and 5 days
Severe/Nonsevere	30/6	29/5	1/1
ICU admission (Yes/No)	1/35	1/33	0/2
Delta days I-II QFT-P	200 (154 to 352)	207 (169 to 359)	68 and 80 days
Delta days I-II TBNK^*c*^	362 (297 to 381)	362 (297 to 381)	NA
Steroids^*d*^ (Yes/No)	34/2	33/1	1 Yes and 1 No
IL-6R Inhibitors^*d*^ (Yes/No)	1/35	1/33	0/2

a QFT-P, QuantiFERON-TB Gold Plus; ICU, intensive care unit; TBNK, peripheral blood T-, B-, and NK-cell assessment; IL-6R, interleukine-6 receptor.

b Quantitative data are represented as median (interquartile range). For the QFT-P positive group, quantitative data are distinctly reported for each of the two subjects, keeping the same order throughout the column.

c T-, B-, and NK-cell reassessment was performed only in 12 subjects after the acute phase of the disease.

d Intravenous steroids and IL-6R inhibitors were administered during hospitalization for COVID-19 after the first sampling for QFT-P and the lymphocyte subset assessment, following the available national guidelines.

A second QFT-P assay was performed at least 43 days after the first assay (median time: 200 days; IQR: 154 to 352 days). All of the QFT-P assays gave an interpretable (determinate) result, with 2 positive (5.6%) and 34 negative (94.4%) tests. The laboratory parameters were reassessed and compared to the corresponding values registered at the time of COVID-19 hospitalization, including the absolute counts of total lymphocytes (*P* < 0.0001) CD3^+^, CD3+CD4^+^, and CD3+CD8^+^. T-lymphocytes were significantly increased (*P* = 0.0005), while the absolute count of neutrophils, the neutrophil to lymphocyte (N/L) ratio, and the d-dimer, fibrinogen, ferritin, and C-reactive protein (CRP) concentrations were significantly reduced (*P* < 0.0001) after hospital discharge. Concerning the QFT-P assay, IFN-γ production in the mitogen and mitogen-nil conditions was significantly increased (*P* < 0.0001), thus allowing for an interpretable result of the test (Table 2).

**TABLE 2:** Laboratory parameters assessed at hospital admission (T0) and after complete recovery (Tpost)^*a*^

Parameter	T0^*b*^	Tpost^*b*^	*P* value
White blood cells (×10^3^/μL)	8.0 (5.1 to 11.3)	6.6 (5.8 to 8.0)	0.0686
Neutrophils (×10^3^/μL)	6.5 (4.2 to 9.5)	3.8 (3.3 to 4.4)	<0.0001
Lymphocytes (×10^3^/μL)	0.7 (0.5 to 1.0)	2.0 (1.6 to 2.3)	<0.0001
N/L Ratio	8.8 (4.9 to 12.8)	1.8 (1.6 to 2.4)	<0.0001
CRP (mg/L)	80.2 (29.2 to 167.1)	1.4 (1.0 to 2.5)	<0.0001
D-dimer (ng/mL)	695.0 (450.5 to 986.5)	227.0 (179.0 to 418.5)	<0.0001
Fibrinogen (mg/dL)	555.0 (495.8 to 690.3)	292.5 (263.0 to 331.3)	<0.0001
Ferritin (ng/mL)	1055.0 (369.0 to 1590.0)	130.0 (43.0 to 211.0)	<0.0001
QuantiFERON-TB Gold Plus			
Mitogen (IFN-γ IU/mL)	0.3 (0.1 to 0.6)	10.0 (10.0 to 10.0)	<0.0001
Mitogen-Nil (IFN-γ IU/mL)	0.1 (0.0 to 0.3)	9.9 (9.9 to 10.0)	<0.0001
T-, B-, NK-cell absolute counts^*c*^			
CD3+ #	433.5 (188.8 to 705.8)	1332.0 (1168.0 to 1796.0)	0.0005
CD3+CD4+ #	275.0 (114.0 to 425.0)	859.5 (706.8 to 1071.0)	0.0005
CD3+CD8+ #	111.5 (68.5 to 177.5)	463.0 (313.0 to 498.8)	0.0005
CD3+CD4+CD8+ #	6.0 (4.0 to 8.8)	21.5 (16.0 to 26.5)	0.0005
CD3+CD4–CD8– #	11.0 (5.3 to 19.0)	47.0 (25.8 to 66.5)	0.0005
CD19+ #	84.0 (61.3 to 108.0)	187.5 (120.3 to 266.5)	0.0024
CD3–CD16+CD56+ #	124.0 (85.0 to 166.5)	216.0 (157.0 to 243.8)	0.0015
CD4/CD8 Ratio	2.2 (1.5 to 3.0)	2.1 (1.7 to 2.8)	0.9097

a N/L ratio, neutrophils to lymphocyte ratio; CRP, C-reactive protein; IFN-γ, interferon gamma; #, absolute count.

b Quantitative data are presented as median (interquartile range); T0: parameters measured at the time of hospitalization for COVID-19; Tpost: parameters measured after complete recovery from COVID-19.

c T-, B-, and NK-cell reassessment was performed on only 12 subjects during the acute phase of the disease (T0) and after complete recovery (Tpost).

In a previous study conducted by our group, we found an increased rate of indeterminate results at the QFT-P assay (22.1%) in COVID-19 hospitalized patients (6) compared to the rates observed in non-COVID-19 patients from the available literature (3.2%) (10) and from our previous experience in different settings (4.3% of results were indeterminate in hematopoietic stem cell transplant recipients from January 2015 to December 2019, unpublished data).

Ward et al. reported that COVID-19 patients with both indeterminate and determinate QFT-P results were lymphopenic, although only subjects in the first group failed to produce an effective IFN-γ response after stimulation (1). Prior work performed by our group confirmed the results observed by Ward et al. and provided evidence that T-lymphocyte reduction in the peripheral blood was correlated with the impairment of IFN-γ production upon phytohemagglutinin (mitogen) stimulation in the QFT-P assay and that this was leading to indeterminate results (6). Our longitudinal series shows that once the acute phase of COVID-19 is resolved, inflammatory markers and peripheral blood leukocyte counts tend to normalize, and effective interferon gamma production by T-lymphocytes is restored after both specific (mycobacterial peptide) and nonspecific (mitogen) stimulation.

One limitation of our study is that the indeterminate results of the QFT-P assay were not confirmed with a second test performed on a different blood sample during the acute phase of COVID-19.

Finally, we observed 2 positive QFT-P assays (5.6%), supporting the importance of retesting patients with indeterminate results in order to identify latent tuberculosis infections (LTBI) and to monitor patients for possible reactivation, considering COVID-19-induced immune-suppression (11, 12).
